# The Cost of Caring: Occupational Stress, Burnout, and Professional Deformation Among Psychiatrists in Georgia

**DOI:** 10.1192/j.eurpsy.2026.12008

**Published:** 2026-07-31

**Authors:** N. Jibuti, L. Jishkariani, K. Jmukhadze

**Affiliations:** Tbilisi State Medical University, Tbilisi, Georgia

## Abstract

**Introduction:**

Occupational stress in psychiatry extends beyond individual strain, progressively reshaping professional identity. Chronic exposure to suffering, systemic deficiencies, and bureaucratic overload often drive psychiatrists toward cynicism, detachment, and diminished empathy—hallmarks of professional deformation. Although widely documented globally, such dynamics remain understudied in Georgia.

**Objectives:**

To assess the prevalence, manifestations, and consequences of occupational stress among psychiatrists in Georgia, with particular focus on how stress undermines empathy and contributes to cynicism and professional erosion.

**Methods:**

A cross-sectional survey was carried out with 52 psychiatrists across Georgia. Data collected included demographics, years of practice, workload perception, empathy changes, stress intensity, symptoms, coping strategies, main stressors, health effects, and career attitudes. Descriptive statistics were used for analysis.

**Results:**

Most respondents were female (86.5%), and the majority had more than 16 years of clinical experience. Workload was reported to increase with seniority by 86.5%. Empathy showed a mixed pattern: 42.3% reported growth, 36.5% described “restructuring” into strict emotional boundaries, and 3.8% reported a decline, suggesting professional detachment. Stress was rated as strong or moderate by 61.6% of participants. The most common symptoms were fatigue (30.8%), reduced patience (23.1%), lower optimism (23.1%), and sleep problems (21.2%). More than half (51.9%) felt their stress had worsened during their careers, showing a cumulative effect on resilience. Main stressors included low pay (76.9%), lack of resources (61.5%), socially deprived patients (48.1%), overcrowded wards (44.2%), and bureaucratic barriers (42.3%). Many used positive coping strategies—social support (86.5%), travel and education (51.9% each), and physical activity (30.8%). However, some reported maladaptive behaviors (7.7%), and most (78.8%) had never sought professional psychological help. Stress had a negative impact on personal life (21.2%) and health (23.1%). Despite these challenges, 86.5% said they would choose psychiatry again, while 13.5% expressed doubt or regret. This shows the coexistence of professional pride with burnout.

**Conclusions:**

Psychiatrists in Georgia experience strong and lasting occupational stress that gradually changes professional identity. While some maintain or even increase empathy, many develop detachment, cynicism, and lower patience—signs of professional deformation. Stress in psychiatry does not only cause exhaustion; it also affects the moral and relational core of the profession. Systemic reforms—better pay, improved resources, fewer bureaucratic burdens, and confidential psychological support—are urgently needed to protect both psychiatrists’ well-being and the quality of patient care.

**Disclosure of Interest:**

None Declared

